# Protein content with parenteral nutrition in critical and hospitalized patients with continuous renal replacement therapies

**DOI:** 10.1186/2197-425X-3-S1-A185

**Published:** 2015-10-01

**Authors:** AV Baez, AM Mañanes, FC Delgado, JLV Gamez, GM Cecilia, FS Gonzalez

**Affiliations:** Hospital Virgen de la Victoria, UCI, Malaga, Spain; Hospital Virgen de la Victoria, Malaga, Spain

## Introduction

ESPEN Guidelines(2009) about parenteral nutrition in critically ill patients recommended protein content of 1,3-1,5 gr/kgr/d (0,208-0,24 gr N) and energy inputs of 20-25 kcal/kgr/d (80-95 kcal non protein /gN). Recent trials detected underline protein content in the most of critical patients and in special cases of CRRT.

## Objectives

Protein content between 2010-2013 to verify trend to higher protein content and diminished non protein calories. In 2013 we compared the differences between protein content of critical ill patients and hospitalized patients and patients with CRRT.

## Methods

We used a Data Base ( NUTRIDATA), registered in the pharmacy of our hospital (550 beds) by case and day. During 2010-2013 protein content and parenteral nutrition bags, gr, grN/kgr and Kcal non protein are registered. In the subgroup of CRRT we registered patients with parenteral nutrition, statistically significance about differences in protein content, we used test of student.

## Results

**Table 1:** Number of cases, number of bags, protein content in bags, grN, grN/kgr and Kcal no prot/grN.

Year	2010	2013
N cases	411	365
N bags	5731	5882
Days/patients	14,2	16,6
GrN	14+_3,28	15,05+_4,23
% grN over 18	12,8	19,6
% grN/kgr over 0,26	15,9	21,3
Kcal no prot/grN	111,62+_22,23	101,82+_25,41
% Kcal no prot/grN	36,8	56,4

**Table 2:** Protein content parenteral nutrition in critically ill patients vs hospitalized patients. Influence of CRRT.

2013	Hospitalized	ICU	CRRT
Number of cases	251	168	53
Number of bags	2973	2178	1015
%GrN between 18-22 Over22	13,56+_3,13 4,4 0,8	15,87+_4,19 20,4 7,4	17,60+_4,28 33,8 13,5
Kcalnoprot/grN %Between 80-95 Over95	112,2+_20 14,5 82,6	97,3+_22,6 44,5 40,6	89,68+_18,4 44,7 27,8
GrN/Kgr % over 0,26	0,204+_0,052 15,6	0,205+_0,056 20,4	0,211+_0,056 22,8

## Conclusions

Increased in protein content between 2010-2013 and diminished calories non proteics /grN. In our hospital we followed the ESPEN Guidelines with important differences in the groups of study and with statistically significant difference.

